# Seasonality of Pneumococcal Nasopharyngeal Carriage in Rural Gambia Determined within the Context of a Cluster Randomized Pneumococcal Vaccine Trial

**DOI:** 10.1371/journal.pone.0129649

**Published:** 2015-07-01

**Authors:** Abdoulie Bojang, James Jafali, Uzochukwu E. Egere, Phillip C. Hill, Martin Antonio, David Jeffries, Brian M. Greenwood, Anna Roca

**Affiliations:** 1 Medical Research Council Unit, Fajara, The Gambia; 2 Centre for International Health, School of Medicine, University of Otago, Dunedin, New Zealand; 3 Faculty of Infectious and Tropical Diseases, London School of Hygiene & Tropical Medicine, London, United Kingdom; Public Health England, UNITED KINGDOM

## Abstract

**Background:**

We conducted an ancillary study among individuals who had participated in a PCV-7 trial in rural Gambia, to determine the influence of season on the prevalence of pneumococcal carriage.

**Methods:**

636 individuals above 30 months of age were followed from 4 to 20 months after vaccination with PCV-7 or meningococcal-conjugate-vaccine. Nasopharyngeal swabs were collected periodically between November 2006 and June 2008. Overall, 4,495 NPS were collected.

**Results:**

Prevalence of pneumococcal nasopharyngeal carriage in the study subjects (median age 11 years) was 55.0%; this prevalence decreased linearly with increasing age (p = 0.001). Prevalence of carriage was significantly higher during the dry than the rainy season for any pneumococcal carriage [57.6% versus 47.8% (p<0.001)], pneumococcal vaccine serotype carriage [10.3% versus 6.5% (p< 0.001)] and non-vaccine serotype carriage [49.7% versus 42.7% (p<0.001)]. Differences remained significant in the adjusted analysis.

**Conclusions:**

In areas of Africa with marked variation in rainfall, seasonality of pneumococcal carriage needs to be considered when interpreting carriage data.

## Introduction

*Streptococcus pneumoniae*, the pneumococcus, is an important cause of pneumonia, meningitis and febrile bacteraemia [1]. In developing countries, including those in sub-Saharan Africa, incidence rates of invasive pneumococcal disease (IPD) are very high, particularly among young children [2–4]. Pneumococcal infections are transmitted by direct contact with respiratory secretions derived from asymptomatic nasopharyngeal carriers as well as from ill patients. Pneumococcal carriage is a necessary step in the progression to disease [5]. In Africa, where rates of IPD are among the highest in the world, the prevalence of carriage is also very high among healthy individuals of all age groups, in both rural and peri-urban areas [6–8].

Prevention of pneumococcal disease is a priority target for reducing infant and child mortality in the developing world. Pneumococcal conjugate vaccines (PCVs) reduce IPD due to serotypes included in the vaccine–vaccine types (VT)–among both vaccinated individuals and their contacts (the latter a consequence of the indirect, herd effect of the vaccine) [9–12]. The indirect effect of the vaccine is driven by a reduction in nasopharyngeal carriage among vaccinated individuals, with a subsequent decrease in transmission of pneumococci in the community [13–16]. In many, but not all situations, a decrease of both carriage and IPD due VT has been followed by an increase in carriage and IPD caused by serotypes that were not represented in the vaccine–non-vaccine types (NVT)—a phenomenon called serotype replacement [17–20].

Pneumococcal conjugates vaccines are currently being deployed widely in sub-Saharan Africa and in other developing countries and carriage studies are being used as early predictors of vaccine impact in different regions, at least to assess their impact on VT. Therefore, it has become increasingly important to strengthen our understanding of factors other than vaccination that can influence trends in pneumococcal colonization as this information is necessary to interpret the results of these impact studies.

The primary aim of the analysis reported in this paper has been to determine the impact of season on pneumococcal carriage in The Gambia, a tropical country with two marked annual seasons—a hot dry season which extends from November to May and a shorter rainy between June and October. For this purpose, we have used the results obtained from nasopharyngeal swabs (NPS) collected over a period of 20 months from a cohort of individuals participating in a community randomized 7-valent PCV (PCV-7) trial in rural Gambia.

## Materials and Methods

### Study population

The study was carried out in Sibanor and the surrounding satellite villages, Western Region, The Gambia. Twenty-one of the 55 villages in the study area were selected with an overall population of 5441 in June 2006. Epidemiological characteristics of the study population have been described previously [8, 21]. A baseline cross-sectional survey showed a high prevalence of pneumococcal nasopharyngeal carriage of approximately 70% [8] before the vaccine was introduced. During the three years of the study annual rainfall ranged between 768mm and 1255mm with the peak rainfall occurring between June and October every year (Hydromet Office, Banjul).

### Trial design

The analysis presented here is based on data collected during a large, cluster-randomized (by village), placebo-controlled trial of PCV-7 conducted to assess the impact of vaccination of the whole community on pneumococcal nasopharyngeal carriage. Details of the study design, the way in which it was conducted and of the overall impact of vaccination have been described previously [13, 22]. In brief, PCV-7 was given to all children below 30 months of age enrolled in the trial and to those born during its course in all study villages. Villages were randomized to two groups. In one group older children and adults received PCV-7 (wholly vaccinated villages) whilst in the other group they received a serogroup C meningococcal conjugate vaccine (partly vaccinated villages) [22]. Vaccination started in July 2006 and continued until 2008 when PCV-7 was introduced as part of the Expanded Programme of Immunization across the whole country.

### Ethical approval

Study participants gave individual written informed consent; written parental consent was obtained for children up to 16 years of age. The study was approved by the joint MRC/Gambia Government Ethics Committee and by the ethics committee of the London School of Hygiene & Tropical Medicine. Conduct of the trial was guided by a Data Safety and Monitoring Board

### Longitudinal study

Six hundred and thirty-six subjects above the age of 30 months at the start of the trial were randomly selected from the 21 study villages for participation in a longitudinal study. Selection of participants was proportional to the number of subjects in each village for the different age groups (2.5 years to less than 5 years, 5 to less than 15 years and 15 years and above). After consent had been obtained, nasopharyngeal swabs (NPS) were collected monthly during the first 3–4 months of follow-up (starting in November 2006) and then every 3 months until June 2008. Therefore, up to 10 NPS could be collected per participant.

### Sample handling

Samples were obtained from the posterior wall of the nasopharynx using a calcium alginate swab and immediately inoculated into vials containing skim milk-tryptone-glucose-glycerol (STGG) transport medium; these vials were placed in a cold box before being transferred to the Medical Research Council Laboratories at Fajara (a distance of 90 km) within eight hours of collection, in accordance with the WHO protocol for evaluation of pneumococcal carriage [23]. Inoculated vials were stored at -70° C until they were tested in batches by subculturing onto gentamicin blood agar (GBA) for selective isolation of *S*. *pneumoniae*.

### Laboratory methods

Isolation and identification of pneumococci was performed as described previously [22]. Serotyping was performed at the MRC Fajara Laboratories, with capsular and factor typing sera (Statens Serum Institute, Copenhagen, Denmark), using a modified latex agglutination assay [24, 25]. Equivocal results were confirmed by the Quellung reaction [26].

### Data management and statistical analysis

Pneumococcal serotypes were grouped as follows: (i) VT: serotypes included in PCV-7 (4, 6B, 9V, 14, 18C, 19F and 23F) and cross-reactive serotype 6A; (ii) NVT: other pneumococcal serotypes not included in the above classification, including non-typeable isolates.

The primary objective of the study was to assess the prevalence of pneumococcal nasopharyngeal carriage (ANY,VT and NVT) between the wet and dry seasons (June to October and November to May, respectively). Pneumococcal nasopharyngeal carriage, of ANY, VT and NVT were represented by dummy variables and analysed separately to accommodate subjects carrying more than one serotype. A sensitivity analysis was conducted with 30 days modification, back and forth, of the dry season definition.

Firstly, summary statistics (median and IQR for the quantitative variables and (n%) for categorical variables) were estimated within each season. Then, their distributions were compared between the two seasons using the Wilcoxon rank-sum test or Chi-square/Fisher’s exact tests, respectively.

Further, logistic regression analysis was applied to quantify the association of pneumococcal carriage with seasonality; reporting odd ratios (OR) and their 95% confidence intervals (95%CI) while adjusting for potential confounders. Both unadjusted and adjusted OR are presented. Estimated OR were adjusted by the a priori chosen potential confounders: age group (<10 versus ≥10 years old) and trial arm (partly versus wholly vaccinated villages). Including other potential confounders such as sex, education levels, occupation, ethnicity, number of samples per subjects and smoking status did not significantly change the estimated odds ratios associated with the two seasons. Presence of significant interactions terms (effect modification) was tested using likelihood ratio tests, but none was statistically significant at α = 0.050.

A multilevel, random intercept, logistic regression modelling technique was applied (using xtmelogit command) to account for the correlations of samples collected from the same subject over time and the clustering of subjects within the same village. However, likelihood ratio tests (though conservative) showed little evidence (P-value >0.1) of within village clustering of subjects. Hence the random intercept models were simplified (using xtlogit command) by ignoring the within village clustering of subjects. There was little evidence of an autocorrelation structure and equal correlation was assumed. Furthermore, robust variance estimator were tested, but gave very similar results to the modelled standard errors.

All the analyses were conducted in Stata 12.1 (StataCorp. Texas, USA). However, all the figures were done in R statistical programing software (R Core Team 2014). P-values <0.05 have been taken to indicate statistical significance

## Results

### Descriptive analysis

Six hundred and thirty-six subjects were followed between November 2006 and June 2008. The median age of study subjects at recruitment was 11 years and 49.2% of participants were males. Demographic and epidemiological characteristics of the study subjects are shown in Table 1.

**Table 1:** Characteristics of the individuals participating in the longitudinal study.

Variable		Number (%)
**Individuals followed**		636
**Individuals per village, median (IQR)**		41 (25, 57)
**Age (years), median (IQR)**		11.0 (4.6, 25.0)
**Age groups (years)**		
	2.5-<5 y	184 (28.9%)
	5-<15 y	208 (32.7%)
	> = 15 y	244 (38.4%)
**Gender**		
	Female	323 (50.8%)
	Male	313 (49.2%)
**Number of years at school**		
	None	307 (48.3%)
	< 1 year	81 (12.8%)
	1–6 years	151 (23.8%)
	7–10 years	72 (11.3%)
	> 10 years	24 (3.8%)
**Able to Read**		
	No	406 (63.9%)
	Yes	229 (36.1%)
**Able to Write**		
	No	430 (67.6%)
	Yes	206 (32.4%)
**Occupation**		
	Farmer	111 (17.5%)
	Housewife	38 (6.0%)
	Student	257 (40.4%)
	Unemployed	117 (18.4%)
	Other	113 (17.8%)
**Smokes**		34 (5.3%)
**Smoker in the household**		
	No	379 (60.1%)
	Yes	252 (39.9%)

**Fig 1:**
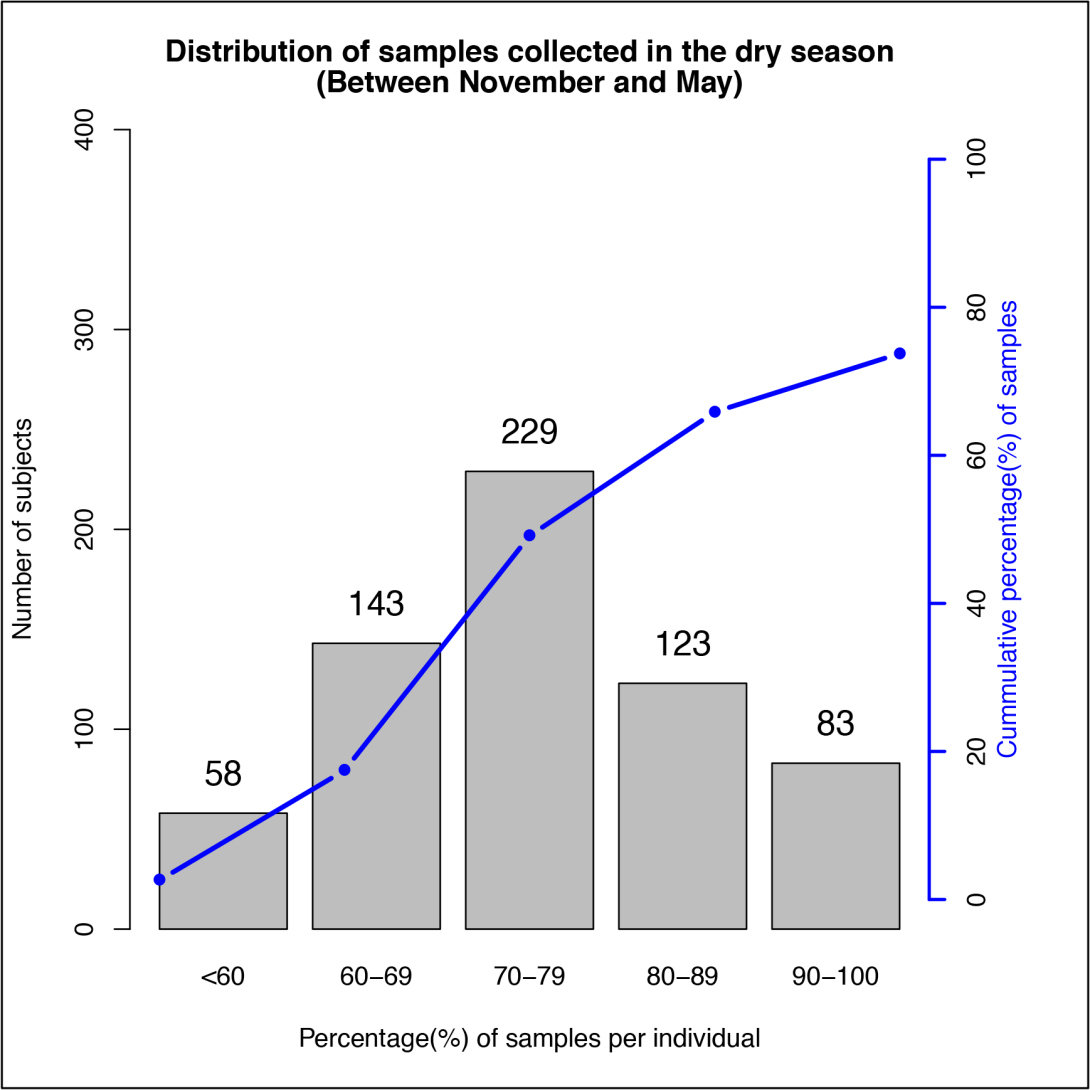
Distribution of samples collected in the dry season. The bar graph shows the distribution of participants based on their percentage of samples collected in dry season. The line graph represents the corresponding cumulative percentage (of the total samples) for the samples collected in the dry season. Interpretations
More subjects, 229(36%), had 70%-79% of their total samples collected in the dry season68%(435/636) of the participants had at least 70% their total samples collected in the dry season.Majority of the participants, 78%(498/363), had 60%-89% of their samples collected in the dry season.

During the follow-up period, 4,495 NPS samples were collected [median 8 samples per individual, IQR 7–9]. Most samples (n = 3,315, representing 73.7% of collected samples) were collected during the dry season. Percentages of samples collected in the dry season for each of the study individuals are shown in Fig 1 and the characteristics of individuals from whom the samples were collected in Table 2.

**Table 2:** Distribution of samples by season.

Factor		Any	Dry	Wet	P-value
**N**		4495	3315	1180	
**Samples, median (IQR)**		8 (7, 9)	6 (5, 7)	3 (2, 3)	<0.001
**Vaccinated**					0.390
	Partially	2002 (44.5%)	1489 (44.9%)	513 (43.5%)	
	Fully	2493 (55.5%)	1826 (55.1%)	667 (56.5%)	
**Baseline age Median(IQR)**		9.0 (4.2, 20.0)	9.2 (4.3, 20.0)	8.6 (4.2, 21.5)	0.410
**Baseline age, n(%)**					0.470
	2.5-<5 y	1465 (32.6%)	1064 (32.1%)	401 (34.0%)	
	5-<15 y	1574 (35.0%)	1173 (35.4%)	401 (34.0%)	
	> = 15y	1456 (32.4%)	1078 (32.5%)	378 (32.0%)	
**Gender**					0.670
	Female	2294 (51.0%)	1698 (51.2%)	596 (50.5%)	
	Male	2201 (49.0%)	1617 (48.8%)	584 (49.5%)	
**Number of years at school**					0.230
	None	1724 (38.5%)	1280 (38.7%)	444 (37.7%)	
	< 1 year	483 (10.8%)	369 (11.2%)	114 (9.7%)	
	1–6 years	1668 (37.2%)	1200 (36.3%)	468 (39.8%)	
	7–10 years	460 (10.3%)	348 (10.5%)	112 (9.5%)	
	>10 years	148 (3.3%)	109 (3.3%)	39 (3.3%)	
**Ability to read**					0.350
	No	2496 (55.6%)	1827 (55.2%)	669 (56.7%)	
	Yes	1995 (44.4%)	1485 (44.8%)	510 (43.3%)	
**Ability to write**					0.067
	No	2878 (64.1%)	2096 (63.3%)	782 (66.3%)	
	Yes	1610 (35.9%)	1213 (36.7%)	397 (33.7%)	
**Occupation**					<0.001
	Farmer	794 (17.7%)	556 (16.8%)	238 (20.2%)	
	Housewife	226 (5.0%)	175 (5.3%)	51 (4.3%)	
	Student	2204 (49.0%)	1615 (48.7%)	589 (49.9%)	
	Unemployed	800 (17.8%)	538 (16.2%)	262 (22.2%)	
	Other	471 (10.5%)	431 (13.0%)	40 (3.4%)	
**Smoker**					0.460
	No	4287 (95.5%)	3158 (95.4%)	1129 (95.9%)	
	Yes	200 (4.5%)	152 (4.6%)	48 (4.1%)	
**Smoker in household**					0.930
	No	2535 (56.6%)	1869 (56.7%)	666 (56.5%)	
	Yes	1940 (43.4%)	1428 (43.3%)	512 (43.5%)	

The overall prevalence of pneumococcal carriage in the study villages during the follow up period was 55.0%; 9.3% for VT carriage and 47.8% for NVT carriage. Prevalence of carriage of both VT and NVT pneumococci decreased with increasing age groups for all serotype groupings (p<0.001). Overall prevalence of pneumococcal carriage was similar in both study groups, although VT carriage was lower, and NVT carriage higher, in wholly vaccinated villages than in partially vaccinated villages (data not shown).

### Seasonality of pneumococcal carriage

**Fig 2:**
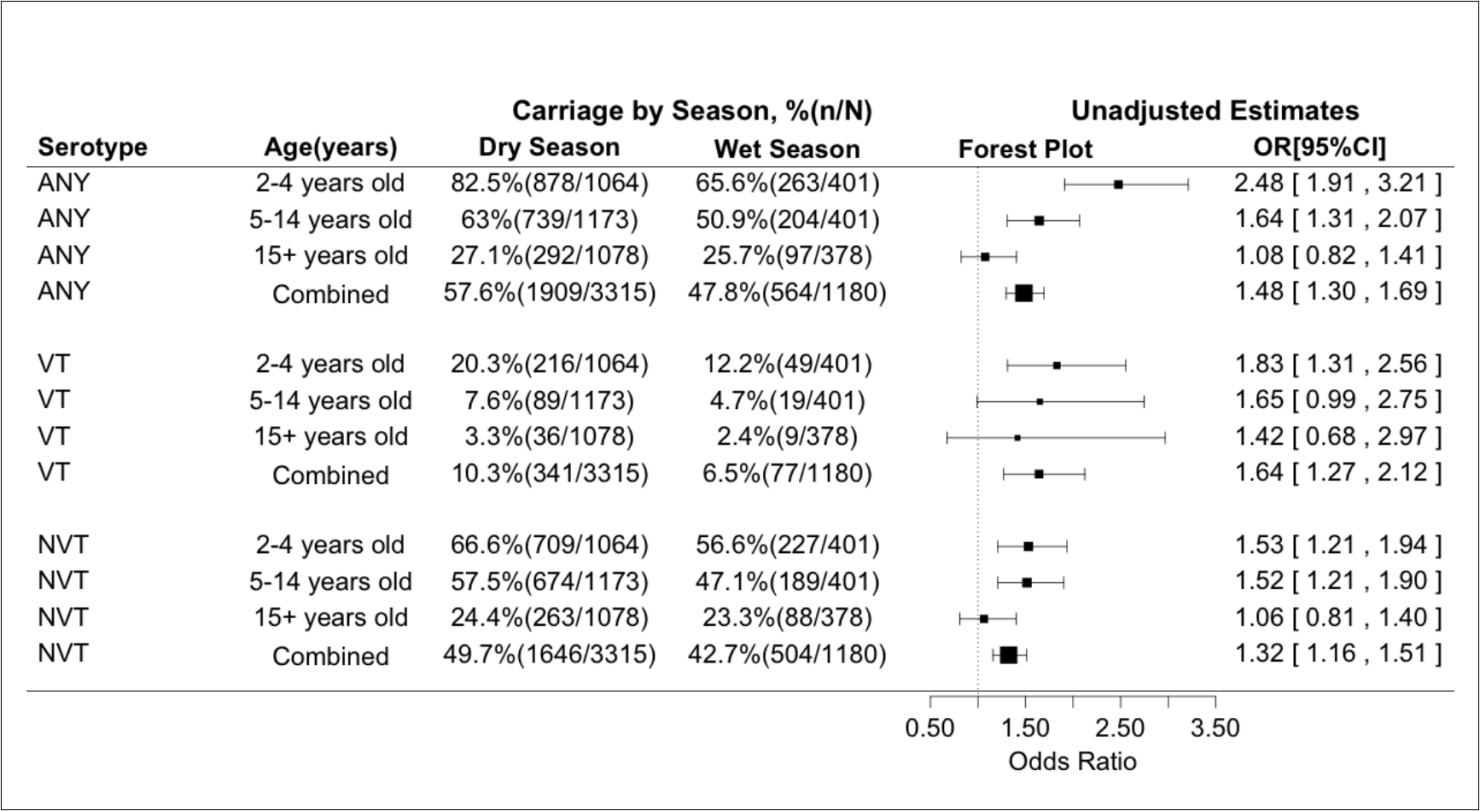
Prevalence of nasopharyngeal carriage in dry and rainy seasons in the study villages by age group for the different study endpoints.

**Fig 3:**
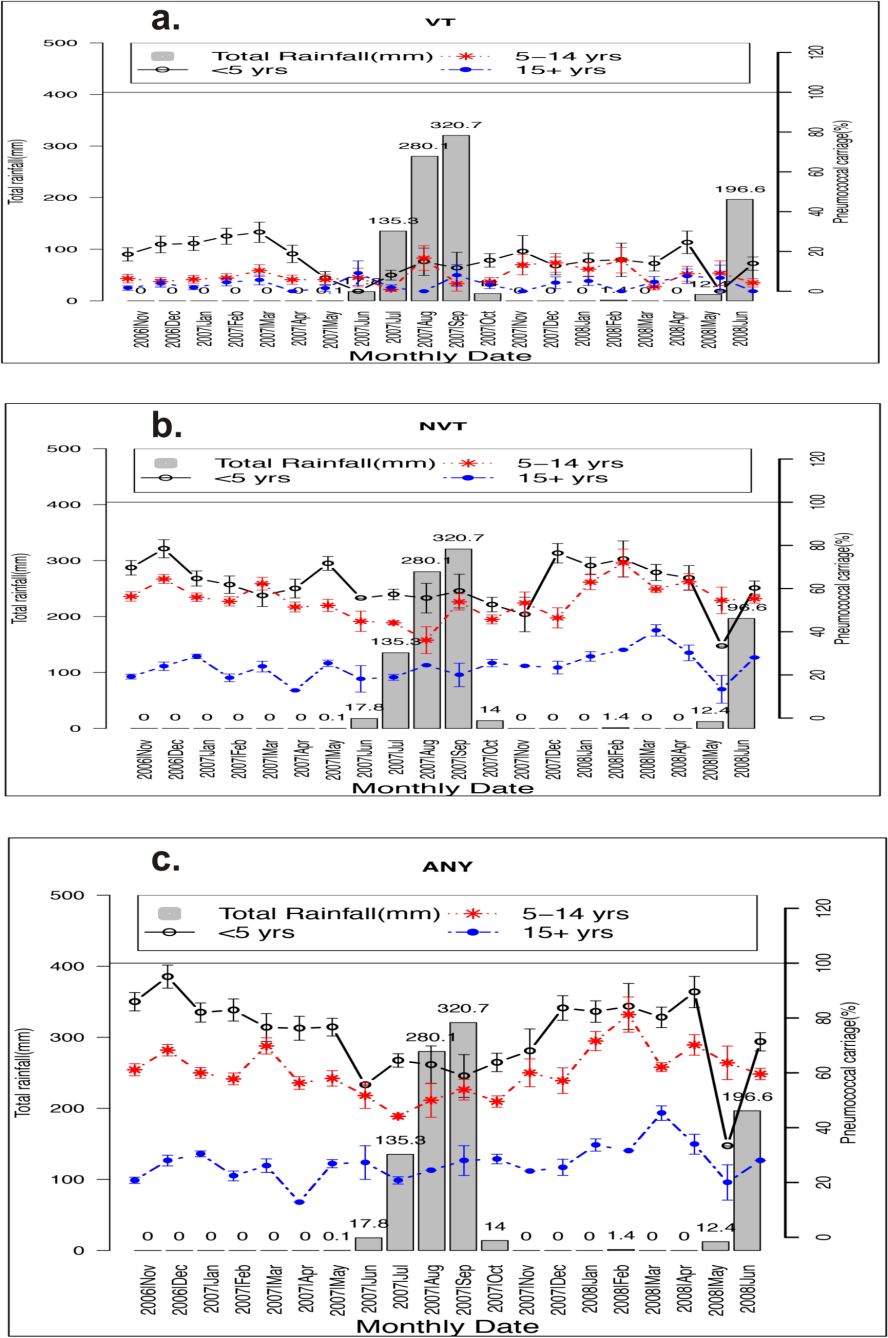
Monthly prevalence of pneumococcal nasopharyngeal carriage among different age groups during the follow-up period; a) VT pneumococcal carriage and b) NVT pneumococcal carriage and c) Any Type pneumococcal carriage.

In the unadjusted analyses, prevalence of pneumococcal carriage was significantly higher during the dry season than during the rainy season for Any carriage [57.6% versus 47.8%, OR = 1.48 95%CI(1.30;1.69), p<0.001], for VT carriage [10.3% versus 6.5%, OR = 1.64 95%CI(1.27;2.12), p<0.001] and for NVT carriage [49.7% versus 42.7%, OR = 1.32 95%CI(1.16;1.51), p< 0.001]. The same trend of higher prevalence of pneumococcal carriage during the dry season was observed in all age groups (Fig 2 and Fig 3).

In the adjusted analyses, prevalence of carriage remained higher during the dry season for Any pneumococcal carriage [OR = 1.87 95%CI(1.58;2.21)], VT carriage [OR = 1.86 95%CI(1.41;2.46)] and for NVT carriage [OR = 1.47 95%CI (1.26;1.72)] (Table 3).

**Table 3:** Adjusted analysis of VT, NVT and Any Type pneumococcal carriage.

Serotype Group	Variables		Unadjusted OR (95%CI)	p-value	*Adjusted OR(95%CI)	p-value
**Any Type**	**Season**					
		Rainy	1		1	
		Dry	1.87(1.58,2.21)	<0.001	1.87(1.58,2.21)	<0.001
	**Age in July 2006, yr**					
		2.5-<5y	1		1	
		5-<15y	0.35(0.26,0.46)	<0.001	0.34(0.25,0.45)	<0.001
		> = 15y	0.06(0.05,0.08)	<0.001	0.06(0.04,0.08)	<0.001
	**Gender**					
		Male	1			
		Female	1.04(0.77,1.40)	0.8	0.84(0.67,1.06)	0.150
**VT**	**Season**					
		Rainy	1		1	
		Dry	1.86(1.40,2.46)	<0.001	1.86 (1.41,2.46)	<0.001
	**Age in July 2006, yr**					
		2.5-<5y	1			
		5-<15y	0.29(0.21,0.41)	<0.001	0.28(0.20,0.40)	<0.001
		> = 15y	0.12(0.08,0.18)	<0.001	0.12(0.08,0.18)	<0.001
	**Gender**					
		Male	1			
		Female	1.16(0.84,1.61)	0.37	0.99(0.74,1.33)	0.940
**NVT**	**Season**					
		Rainy	1		1	1
		Dry	1.46(1.25,1.71)	<0.001	1.47 (1.26,1.72)	<0.001
	**Age in July 2006, yr**					
		2.5-<5y	1			
		5-<15y	0.63(0.49,0.08)	<0.001	0.62(0.49,0.79)	<0.001
		> = 15y	0.13(0.10,0.17)	<0.001	0.13(0.10,0.16)	<0.001
	**Gender**					
		Male	1			
		Female	1.01(0.79,1.30)	0.92	0.86(0.70,1.05)	0.150

## Discussion

This paper reports an extensive study of the seasonality of pneumococcal nasopharyngeal carriage. The data were collected as part of a cluster randomized trial conducted in rural Gambia, an area with a high prevalence of pneumococcal nasopharyngeal carriage. We evaluated the prevalence of pneumococcal carriage in a cohort of individuals aged 2.5 years or more, some of whom had been vaccinated previously with PCV-7. Our main findings were that pneumococcal carriage increased during the dry season for all age groups regardless of PCV-7 vaccination status.

Prevalence of pneumococcal carriage oscillated widely over the follow up period in all age groups. When the analysis was stratified by season (dry versus rainy), consistent results were obtained for the different pneumococcal serotype groups and different age groups, with carriage being higher in the dry season. Although this study only considered subjects aged 30 months and above, it is likely that the observed seasonality would be the same among younger age groups. Findings of this study are similar to a previous smaller report on pneumococcal carriage [27] in an area approximately 150 km from this study location where infant mother pairs were sampled during infancy; pneumococcal nasopharyngeal carriage was higher during the dry season for both mothers and babies. As part of the PCV-7 trial, a larger group of subjects in whom the impact of vaccination of the whole or part of the community was assessed [22], post-vaccination cross-sectional surveys were conducted at different calendar period. The highest overall pneumococcal carriage rate was found in the third survey conducted during the late dry season and the lowest in the second conducted during the rainy season [22] in accordance with the data presented here.

Data obtained from several studies of IPD in The Gambia are also in line with the seasonality in pneumococcal carriage that we have observed, supporting the view that higher prevalence of nasopharyngeal carriage at a community level goes along with higher incidence of disease. Incidence of IPD (including pneumococcal meningitis) among young Gambian children participating in a 9-valent PCV trial was highest during the dry season [28]. In a hospital-based study undertaken in the capital Banjul, cases of pneumococcal meningitis were concentrated during the last half of the dry season with a pattern similar to meningococcal disease [29]. Seasonality of IPD has also been seen elsewhere in the Sahel and sub-Sahel including Burkina Faso, Nigeria, Ghana, Kenya and Mali [30–34] with the highest incidence being during the dry season. The similar seasonality patterns between carriage and IPD indicate that disease increases when transmission increases. Similar findings have been observed among the Navajo population in the USA where an increase in both IPD and carriage occurs during autumn. Interestingly, in the latter study, the incidence of pneumococcal pneumonia peaked after the peak of IPD and carriage [35]. In a recent commentary [36] van Hoek and Miller noted that no clear association between seasonality of pneumococcal carriage and IPD is found unless disease is split into pneumonia and non-pneumonia presentations. For non-pneumonia presentation, there was evidence for association with an increase in carriage prevalence but this was not the case for bacteraemic pneumonia for which seasonal variation was associated with Respiratory Syncitial Virus activity.

Why carriage and IPD should be more prevalent in the dry season than in the rainy season is not certain. It could be due to social factors such as more intermixing during the dry season when less farm work needs to be done and the time when children–the drivers of pneumococcal transmission at a community level [22]—attend school. However, it is also likely that climatic factors play a direct role with exposure to pneumococci being more likely to lead to carriage during the dry time of the year and then to cause more disease. A similar seasonal pattern is seen for meningococcal disease in countries of the African ‘meningitis belt’ which includes The Gambia. In this case the climatic factor most closely linked to disease incidence is absolute humidity and this could also be the case for the pneumococcus [37].

The seasonality of clinical pneumonia in The Gambia shows a different seasonal pattern with a higher incidence during the rainy season [29, 38] which suggests the role of viruses during the wet and humid season [39] and possibly enhanced misdiagnosis because the clinical presentation of pneumonia overlaps with that of malaria which is most prevalent during these months [40–42].

Interpretation of our main results should be treated with caution as our study has some limitations. Firstly, the interpretation of seasonality is based on less than 2 years of follow-up. The reason for this short period of follow-up was mass administration of azithromycin in the study area as part of the Gambian National Trachoma Elimination campaign [22] which had a major impact on pneumococcal carriage. Samples collected after the campaign started were not included in this seasonality analysis resulting in a follow up period of less than 2 years. Secondly, the majority of samples included in the study were collected during the dry season. However, this imbalance should not affect our conclusions as the number of samples was large and the characteristics of individuals studied in each season were similar. Despite these limitations the plausibility of our results is supported by the demonstration of similar seasonal trends in carriage and invasive pneumococcal studies in previous studies [22, 27–30, 43] as described above. In addition, this is an add-on study of the trial and therefore no formal power analysis was conducted.

Investigation of changes in the pattern of pneumococcal colonization provides a straightforward way of measuring the impact of PCVs on VT which may be especially useful in countries which do not have the resources to establish long-term surveillance for IPD. Our results underline the importance of considering the potential of external factors, including seasonality, when designing impact studies and interpreting their results. Understanding the factors that influence asymptomatic pneumococcal carriage is necessary to fully interpret the impact of external interventions on pneumococcal disease.
